# Drug reformulations and repositioning in the pharmaceutical industry and their impact on market access: regulatory implications

**DOI:** 10.3402/jmahp.v2.22813

**Published:** 2014-01-29

**Authors:** Susana Murteira, Aurélie Millier, Zied Ghezaiel, Michel Lamure

**Affiliations:** 1 University of Lyon, University Claude Bernard Lyon I, UFR d'Odontologie, 11 rue Guillaume Paradin, 69372, Lyon, Cedex 08, France; 2 Lundbeck SAS, 37-45, Quai du Président Roosevelt, 92445 Issy-les-Moulineaux, Cedex, Paris, France; 3 Creativ-Ceutical S.A., 215, rue du Faubourg St-Honoré 75008, Paris, France

**Keywords:** repurposing, reformulation, repositioning, regulation, regulatory, market access

## Abstract

**Background:**

Repurposing has become a mainstream strategy in drug development, but it faces multiple challenges, amongst them the increasing and ever changing regulatory framework. This is the second study of a series of three-part publication project with the ultimate goal of understanding the market access rationale and conditions attributed to drug repurposing in the United States and in Europe. The aim of the current study to evaluate the regulatory path associated with each type of repurposing strategy according to the previously proposed nomenclature in the first article of this series.

**Methods:**

From the cases identified, a selection process retrieved a total of 141 case studies in all countries, harmonized for data availability and common approval in the United States and in Europe. Regulatory information for each original and repurposed drug product was extracted, and several related regulatory attributes were also extracted such as, designation change and filing before or after patent expiry, among others. Descriptive analyses were conducted to determine trends and to investigate potential associations between the different regulatory paths and attributes of interest, for reformulation and repositioning cases separately.

**Results:**

Within the studied European countries, most of the applications for reformulated products were filed through national applications. In contrast, for repositioned products, the centralized procedure was the most frequent regulatory pathway. Most of the repurposing cases were approved before patent expiry, and those cases have followed more complex regulatory pathways in the United States and in Europe. For new molecular entities filed in the United States, a similar number of cases were developed by serendipity and by a hypothesis-driven approach. However, for the new indication's regulatory pathway in the United States, most of the cases were developed through a hypothesis-driven approach.

**Conclusion:**

The regulations in the United States and in Europe for drug repositionings and reformulations allowed confirming that repositioning strategies were usually filed under a more complex regulatory process than reformulations. Also, it seems that parameters such as patent expiry and type of repositioning approach or reformulation affect the regulatory pathways chosen for each case.

## Introduction

Repurposing has become a mainstream strategy in drug development (1, 2). Repurposing a molecule consists of finding new therapeutic uses for already known drugs, developing different formulations for the same drug, or creating new combinations of at least two drugs previously used as separate drug products. Those repurposing strategies are referred to as ‘repositioning’, ‘reformulation’, and ‘combination’, respectively (1).

Drug repurposing with its different strategies faces multiple challenges; these may include clinical related issues, such as the need to run new trials and start from scratch when the data from the preclinical or clinical studies for the original product are outdated and therefore don't meet actual regulatory requirements (2). In terms of regulatory environment, drug reformulation in Europe, for example, faces an increasingly stricter regulatory environment, with several initiatives taken since the early 2000s to regulate some of the pharmaceutical industries’ drug protection tactics (3). Additionally, some reformulation types such as the enantiomeric switch were matched by a regulatory response that increasingly makes these strategies less interesting to developers. Current regulations on this topic oblige the sponsors to recognize the occurrence of chirality when developing a new drug and encourage them to separate the different stereoisomers, assess their profile, and make a rational selection from the early development stages onwards (3, 4).

This is the second study of a series of a three-part publication project with the ultimate goal of understanding the rationale of the market access outcome and conditions attributed to drug reformulation and repositioning in the pharmaceutical industry in the United States and in Europe. In the first study, we have proposed a harmonized nomenclature and taxonomy for repurposing strategies (1); with this second study, we will evaluate the regulatory path associated with each type of repurposing strategy; and, with the last study of this series, we will try to identify the determinants of successful market access outcome for repurposed drugs in the United States and in Europe (5).

The aim of the current study is to understand the regulatory implications for the different repurposing strategies. As such, through this study, we propose to evaluate the regulatory path associated with each type of repurposing strategy, in accordance with the previously proposed nomenclature.

The next step will be to describe the pricing and reimbursement trends for repurposing case studies and evaluate the parameters that may contribute to a positive pricing and reimbursement decision (5).

## Methods

The systematic literature review presented in the first article of this series allowed retrieving a list of 87 repurposed drugs in the United States, France, the United Kingdom, and Germany (corresponding to 125 cases when duplicates were counted – a drug may be repurposed several times).

A case-by-case review was performed in order to identify the types of repurposing (repositioning, reformulation, or combination). Conformity to the definitions of repurposing strategies was assessed by two independent reviewers, and disagreements were solved by consensus. Cases that did not meet the definitions’ criteria were excluded and are not presented in the present article.

### Regulatory environment

Regulatory considerations are key determinants for the development of drug repurposing strategies (6). Regulatory frameworks in the United States and in Europe, including application types, submission pathways, and eventual exclusivity benefits that might be related to drug repurposing, are as follows:

1. In the United States, new drug applications (NDAs) are classified into different chemical types (e.g., NDA type 1 for new molecular entities), and the drug application can be filed according to one of the possible regulatory paths, namely, section 505(b)(1), section 505(b)(2), and section 505(j) (6–9). Table I provides an overview of the classification types for a new drug application and the US Food and Drug Administration (FDA) review classification that can be applicable (priority, standard, or orphan drug review).
  To make minor changes (label, new dosage or strength, etc.) in a product that already has an approved NDA (or BLA for biologic products), a company must submit a supplemental new drug application (sNDA) (or sBLA for biologic drugs) (10–12).
2. In Europe, Directive 2001/83/EC [particularly articles 6, 8 (3), 10 (3), and 10 (5)] as amended by Directive 2004/27/EC, provides the main legal basis for drug applications for repurposed drugs. Additionally, the application process in Europe can be filed via the centralized, decentralized/mutual recognition or national procedure (13).

Regarding market exclusivity benefits and intellectual property implications (in the United States and European Union), repurposing can offer valuable exclusivity for the new product by protecting its new formulation, indication, or methods of use. This is true even if the original product has already lost its active pharmaceutical ingredient (API), formulation, and/or indications’ patent protection (14). Table II provides a summary of the incentives and exclusivity protection possibilities that can be offered to a repurposed product in the United States and European Union.

**Table I:** List of some NDA chemical types and FDA review classifications (7)

NDA chemical types		

Number	Chemical type	Meaning
1	New molecular entity (NME)	An active ingredient that has never been marketed in the United States
2	New active ingredient or new derivative	A chemical derived from an active ingredient already marketed
3	New dosage form	A new dosage form or new formulation of an active ingredient already on the market
4	New combination	A drug that contains two or more compounds, the combination of which has not been marketed together in a product
5	New formulation or new manufacturer	A product that duplicates another company's already marketed drug product
6	New indication	A new use for a drug product already marketed by a different company
FDA review classifications		

Letter	Review classification	Meaning

P	Priority review drug	A drug that appears to represent an advance over available therapies
S	Standard review drug	A drug that appears to have therapeutic qualities similar to those of an already marketed drug
O	Orphan drug	A product for which the sponsor received orphan designation under the Orphan Drug Act (ODA)

**Table II:** Potential exclusivity and incentives granted by the US FDA and European Commission for repurposed products (15)

	United States	European Union
Data exclusivity	3 years	1 year for well-established drugs and biologics
	5 years for NMEs	8 years for full dossiers as per Article 6 of Directive 2001/83/EC
Incentives for pediatric studies	6-month exclusivity extension	6-month SmPC extension
		10-year market protection for PUMAs
Incentives for orphan drugs	7-year market protection	10-year market protection
Combined orphan and pediatric incentives	7-year, 6-month market protection	12-year market protection

NME: new molecular entity; SmPC: summary of product characteristics; PUMA: pediatric-use marketing authorization.

### Approval history and regulatory information

Information regarding approval history data was identified and collected for each case. In each country, the regulatory agency website was used, as well as other sources, as presented in Table III.

**Table III:** Sources for regulatory and market approval history

Scope	Source	Link
Global	Medtrack	http://v1.medtrack.com/research/defaultasp
European Union	European Medicines Agency (EMA)	http://www.ema.europa.eu/ema/index.jsp?curl=pages/medicines/landing/epar_search.jsp&mid=WC0b01ac058001d124
France	Agence Nationale de Sécurité du Médicament et des Produits de Santé (ANSM)	http://ansm.sante.fr/
	Thériaque	http://www.theriaque.org/apps/recherche/rch_simple.php
	La Haute Autorité de Santé (HAS)	http://www.has-sante.fr/
	E-Vidal	http://www.evidal.net/index.php
Germany	Bundesinstitut für Arzneimittel und Medizinprodukte (BfArM)	http://www.bfarm.de
	FachInfo	http://www.fachinfo.de/
	Gemeinsame Bundesausschuss (G-BA)	http://www.g-ba.de/
United Kingdom	Medicines and Healthcare Products Regulatory Agency (MHRA)	www.mhra.gov.uk
	Electronic Medicines Compendium (EMC)	http://www.medicines.org.uk/emc/
	National Institute for Health and Care Excellence (NICE)	http://www.nice.org.uk/
	Scottish Medicines Consortium (SMC)	http://www.scottishmedicines.org.uk/SMC_Advice/Advice_Directory/SMC_Advice_Directory (UK launch date)
United States	Drugs@FDA	http://www.accessdata.fda.gov/scripts/cder/drugsatfda/index.cfm

Once all reports, databases, and websites were identified, we developed a standardized data extraction grid to collect approval and regulatory information for each original and repurposed drug product. We also extracted several attributes related to repurposing.

#### Designation change

We assessed whether the source and the target product had an orphan designation (i.e., a condition affecting fewer than 66 persons in a population of 100,000 in the United States, or affecting fewer than 50 persons in a population of 100,000 in the European Union). A four-category variable was then constructed (‘orphan to orphan’: from orphan to orphan; ‘non-orphan’: from orphan to non-orphan; ‘orphan’: from non-orphan to orphan; and ‘no change’: from non-orphan to non-orphan).

#### Approval time

The year of approval was collected for each repurposing case and categorized according to the period of approval (before 1999, between 1999 and 2008, or after 2008). These date cut-offs were chosen because these were the dates after which several regulations in Europe pertaining to drug registration and pricing changed, which could have had a potential impact on the type of repurposed drugs approved and regulatory path used.

#### Patent expiry and marketing authorization approval

We assessed whether the repurposed drug (target product) was approved before or after the patent expiry of the source product, and whether the target product was approved before or after marketing authorization approval of the source product.

#### Company and brand name

We assessed whether there was any change in the company and in the brand name of the target product compared to source product.

#### Repositioning approach

For repositioning cases, we assessed whether the approach used was serendipity (fortuitous discovery of a target product indication) or hypothesis-driven (the discovery of the new indication relies on understanding of the disease physiopathology and/or associated drug mechanism).

#### Reformulation group

For reformulation cases, we evaluated the reformulation group, as defined in the first article of the series (1). Group 0 referred to chiral switch and other switches, group 1 to modified-release formulations, group 2 to new pharmaceutical forms, and group 3 to new administration routes.

## Statistical analysis

### Selection of cases

Since the goal of this study is to compare the trends of regulatory pathways per repurposing type and per geographical area, we have decided to create a selection algorithm in order to eliminate any potential bias due to insufficient or heterogeneous data and, thus, to allow a more robust interpretation of the results. Cases were selected based on several criteria, including geographical scope, data availability, and repurposing history, in order to choose up to two or three cases per classification type. The following prioritization system was used in order to eliminate heterogeneity as much as possible:

1. Cases with wider geographical scope are preferred (all countries>USA +2EU>3EU>USA+1EU>2EU>USA>1EU) in order to allow research for geographical trends.
2. Cases with appropriate data availability are preferred (available details on original and new products) in order to evaluate the impact on the repurposed *versus* original product.
3. Cases with less consecutive repurposing history are preferred (repositioned or reformulated once>repositioned once and reformulated once>repositioned and/or reformulated more than once) in order to allow one to analyses the effect of the initial repurposing strategy.

### Description of cases

On a country basis, the distribution of repurposing type and regulatory application type was described for each of the repurposing cases included in the analysis.

### Description of regulatory paths and association between regulatory paths and attributes

The distribution of cases according to application type was presented, and descriptive analyses were conducted to determine trends and to investigate potential associations between the different regulatory paths and attributes of interest, for reformulation and repositioning cases separately.

### Statistical considerations

All statistical analyses were performed using SAS 9.3 software (SAS Institute, Cary, NC).

## Results

### Description of cases

As previously mentioned, 87 drugs that went through repurposing were identified in the United States, France, the United Kingdom, and Germany (corresponding to 125 case studies when duplicates were counted, i.e., the same drug was repurposed several times). Out of these 125 cases, the proposed nomenclature for drug repurposing could not be applied to 20 cases: five were repositioned in the same therapeutic area, two were reformulated drugs using active metabolites, and one was a combination drug that was not approved. The proposed definition was in accordance with the original type of repurposing (as reported in the literature) for all cases except for nine cases that were edited. Finally, each case was split into several cases when there were geographical duplicates. The pre-final list contains 131 repurposing cases.

### Cases selection and classification

**Fig. 1:**
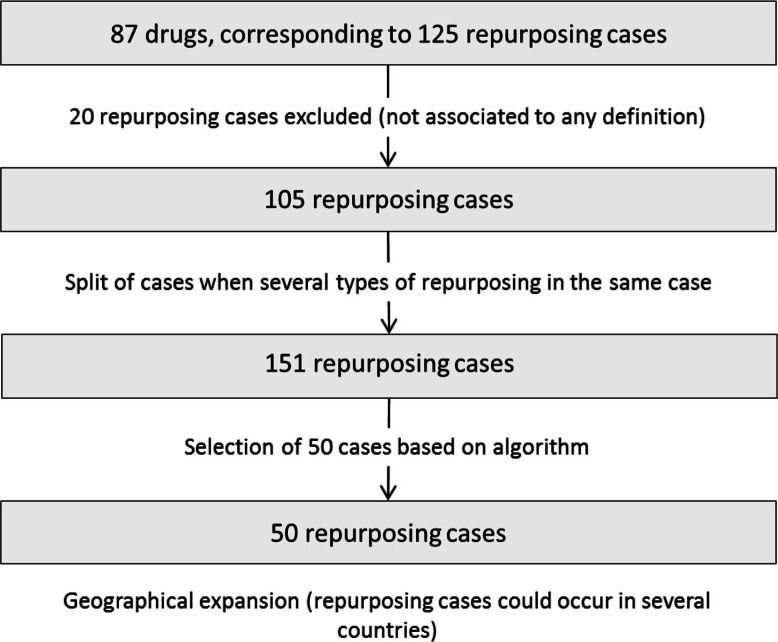
Repurposed cases: selection process.

The application of the selection algorithm for elimination of heterogeneity parameters concerning availability of geographical geographical data, comparative original and repurposed product data, and repurposing strategy data led to a final list of 50 selected repurposing cases that were used for further analysis. Figure 1 describes this selection process.

**Fig. 2:**
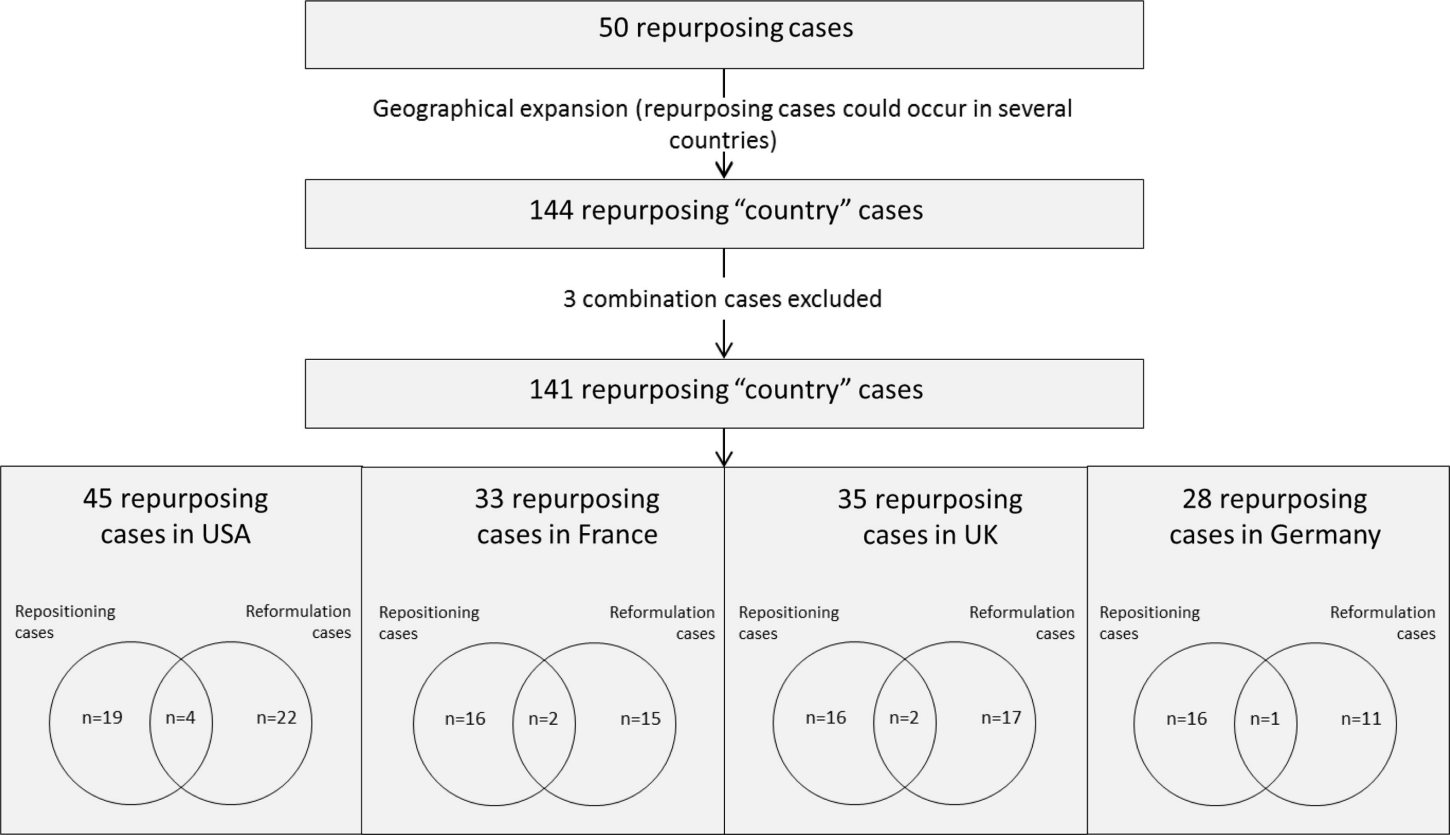
Repurposed cases: geographical distribution.

As a same repurposing case could occur in several countries, geographical duplicates were expanded (Figure 2). The final list contains 144 ‘country’ cases of repurposing, among which three combinations were excluded from the analysis. This is decomposed into 45 repurposed cases in the United States, 33 in France, 35 in the United Kingdom, and 28 in Germany.

Table IV presents the application types that are associated with the cases of each country, as well as the distribution of repurposing types, for reformulation and repositioning cases (excluding combinations).

**Table IV:** Description of regulatory paths

	All repurposing cases*	Reformulation**	Repositioning**
United States	*N*=45	*N*=26	*N*=23
Missing values	0	0	0
NDA type 1 section 505(b)	7 (15.6%)	0	7 (30.43%)
NDA type 2 section 505(b)	2 (4.4%)	2 (7.69%)	0
NDA type 3 section 505(b)	16 (35.6%)	14 (53.85%)	5 (21.74%)
NDA type 3 section 505(b)(2)	4 (8.9%)	3 (11.54%)	1 (4.35%)
NDA type 4 section 505(b)	1 (2.2%)	1 (3.85%)	1 (4.35%)
NDA type 5 section 505(b)	2 (4.4%)	1 (3.85%)	1 (4.35%)
NDA type 5 section 505(b)(2)	2 (4.4%)	2 (7.69%)	0
NDA type 6 section 505(b)	1 (2.2%)	0	1 (4.35%)
sNDA (new indication)	5 (11.1%)	0	5 (21.74%)
sNDA (new formulation)	1 (2.2%)	1 (3.85%)	0
sBLA	4 (8.9%)	2 (7.69%)	2 (8.70%)
France	*N*=33	*N*=17	*N*=18
Missing values	0	0	0
National	12 (36.4%)	9 (52.94%)	4 (22.22%)
Mutual recognition	5 (15.1%)	3 (17.65%)	3 (16.67%)
Centralized	16 (48.5%)	5 (29.41%)	11 (61.11%)
United Kingdom	*N*=35	*N*=19	*N*=18
Missing values	0	0	0
National	18 (51.4%)	14 (73.68%)	6 (33.33%)
Mutual recognition	0	0	0
Centralized	17 (48.6%)	5 (26.32%)	12 (66.67%)
Germany	*N*=28	*N*=12	*N*=17
Missing values	1 (3.5%)	0	1 (5.9%)
National	10 (35.7%)	7 (58.3%)	4 (23.5%)
Mutual recognition	0	0	0
Centralized	17 (60.7%)	5 (41.7%)	12 (70.6%)

* Excluding combinations.

** Note that the number of reformulation cases added to the number of repositioning cases is greater than the total number of repurposed cases, as some cases were both reformulation and repurposing cases.

NDA, new drug application; sBLA, supplemental biologic license application.

In the United States, the distribution of the case studies' application type by repurposing strategy shows that ‘NDA chemical types’ type 1 (new molecular entity), type 6 (new indication), and sNDA (new indication) were solely used for drug-repositioning cases. NDA chemical type 2 (change in an active ingredient, e.g., chiral switch), type 5 (2) (new formulation or new manufacturer), and sNDA (new formulation) were exclusively used for reformulations. Other chemical types, such as NDA types 3 and 4, were used for both reformulation and repositioning cases. Regarding sBLAs, both reformulations and repositioning have used this regulatory process (6).

Within the studied European countries, most of the applications for reformulated products were filed through a national application. In contrast, for repositioned products, the centralized procedure was the most frequently used regulatory pathway. Interestingly, the mutual recognition procedure was used only in France and was evenly used for reformulation and repositioning cases.

We evaluated the correspondence between the regulatory pathways in the European and US repurposed cases. In order to do so, we have selected the case studies that were simultaneously approved in the United States and in at least one of the European countries studied, and we evaluated the trends between those two regions (Table V). From the common repositioned cases (*n=*16) and reformulated case studies (*n=*15), an equivalence does not seem to exist between the type of application used in the United States and the type used in Europe. Indeed, to the same type of regulatory application in the United States corresponded several or different applications types in Europe (national, centralized or mutual recognition), regardless of the type of application or repurposing strategy studied.

**Table V:** Comparative overview of application types per country (for case studies simultaneously in the United States and Europe)

			Application type			
			
Case study repositionings	Original indication	New indication	United States (*N*=23)	France (*N*=18)	United Kingdom (*N*=18)	Germany (*N*=17)
Duloxetine	Major depression disorder (MDD)	Generalized anxiety disorder (GAD)	sNDA (new indication)	Centralized	Centralized	Centralized
Imatinib	Chronic myeloid leukemia (CML)	Gastrointestinal stromal tumor (GIST)	sNDA (new indication)	Centralized	Centralized	Centralized
Imatinib	CML, GIST	Myelodysplastic/myeloproliferative diseases, metastatic dermatofibro-sarcoma protuberans (MDS/MPD, DFSP)	sNDA (new indication)	Centralized	Centralized	Centralized
Propranolol	Angina, hypertension	Migraine	sNDA (new indication)	National	National	–*
Methotrexate	Cancer	Rheumatoid arthritis (RA)	sNDA (new indication)	National	–	National
Rituximab	Non-Hodgkin's lymphoma (NHL)	RA	sBLA	Centralized	Centralized	Centralized
Botulinum Toxin A	Blepharospasm, cervical dystonia	Axillary hyperhidrosis	sBLA	National	National	National
Bosentan Monohydrate	Treatment of congestive heart failure (intended)	PAH	NDA type 1 section 505(b)	Centralized	Centralized	Centralized
Crizotinib	Anaplastic large-cell lymphoma (intended)	Non-small cell lung cancer (NSCLC)	NDA type 1 section 505(b)	Centralized	Centralized	Centralized
Galantamine	Post-polio paralysis and neuropathic pain (intended)	Alzheimer's disease	NDA type 1 section 505(b)	Mutual recognition	National	National
Plerixafor	Human immunodeficiency virus (HIV) (intended)	Mobilization of hematopoietic stem cell	NDA type 1 section 505(b)	Centralized	Centralized	Centralized
Sildenafil Citrate	Angina (intended)	ED ‘Viagra’	NDA type 1 section 505(b)	Centralized	Centralized	Centralized
Minoxidil	Hypertension, oral formulation	MPB, topical formulation	NDA type 3 section 505(b)	National	National	National
Retinoic acid/Tretinoin	Acne (topical gel)	Acute promyelocytic leukemia (APL) (capsules)	NDA type 3 section 505(b)	Mutual recognition	National	–
Sildenafil Citrate	Angina (intended), erectile dysfunction (ED) ‘Viagra’	Pulmonary arterial hypertension (PAH) ‘Revatio’	NDA type 5 section 505(b)	Centralized	Centralized	Centralized
Finasteride	Benign prostatic hyperplasia (BPH)	Male pattern baldness (MPB)	NDA type 3 section 505(b)	Mutual recognition	National	–

* Not approved.

### Association between regulatory paths and attributes in repositioning cases

Table VI presents the distribution of the different regulatory application types and pathways according to their repositioning-related attributes, for each country.

**Table VI:** Distribution of regulatory application type by classification types within repositioning cases

	Designation change				Approval time			Marketing authorization		Patent expiry		Company		Brand name		Repositioning approach	
							
Application type	No change	Orphan drugs	Non orphan	Orphan to orphan	Before 1999	1999–2008	After 2008	Before MA	After MA	Before PE	After PE	Same company	Different company	Same brand	Different brand	Serendipity	Hypothesis-driven
United States	13	5	2	3	8	11	4	7	16	16	7	17	6	14	9	9	14
NDA type 1 section 505(b)	3 (23.1%)	3 (60.0%)	0	1 (33.3%)	2 (25.0%)	3 (27.3%)	2 (50.0%)	7 (100.0%)	0	6 (37.5%)	1 (14.3%)	6 (35.3%)	1 (16.7%)	6 (42.9%)	1 (11.1%)	3 (33.3%)	4 (28.6%)
NDA type 2 section 505(b)	0	0	0	0	0	0	0	0	0	0	0	0	0	0	0	0	0
NDA type 3 section 505(b)	4 (30.8%)	1 (20.0%)	0	0	4 (50.0%)	0	1 (25.0%)	0	5 (31.2%)	3 (18.8%)	2 (28.6%)	2 (11.8%)	3 (50.0%)	0	5 (55.6%)	3 (33.3%)	2 (14.3%)
NDA type 3 section 505(b)(2)	1 (7.7%)	0	0	0	0	0	1 (25.0%)	0	1 (6.2%)	0	1 (14.3%)	0	1 (16.7%)	0	1 (11.1%)	0	1 (7.1%)
NDA type 4 section 505(b)	1 (7.7%)	0	0	0	0	1 (9.1%)	0	0	1 (6.2%)	0	1 (14.3%)	0	1 (16.7%)	0	1 (11.1%)	0	1 (7.1%)
NDA type 5 section 505(b)	1 (7.7%)	0	0	0	0	1 (9.1%)	0	0	1 (6.2%)	1 (6.2%)	0	1 (5.9%)	0	0	1 (11.1%)	0	1 (7.1%)
NDA type 5 section 505(b)(2)	0	0	0	0	0	0	0	0	0	0	0	0	0	0	0	0	0
NDA type 6 section 505(b)	0	1 (20.0%)	0	0	0	1 (9.1%)	0	0	1 (6.2%)	1 (6.2%)	0	1 (5.9%)	0	1 (7.1%)	0	1 (11.1%)	0
sNDA (new indication)	3 (23.1%)	0	0	2 (66.7%)	2 (25.0%)	3 (27.3%)	0	0	5 (31.2%)	3 (18.8%)	2 (28.6%)	5 (29.4%)	0	5 (35.7%)	0	1 (11.1%)	4 (28.6%)
sNDA (new formulation)	0	0	0	0	0	0	0	0	0	0	0	0	0	0	0	0	0
sBLA	0	0	2 (100.0%)	0	0	2 (18.2%)	0	0	2 (12.5%)	2 (12.5%)	0	2 (11.8%)	0	2 (14.3%)	0	1 (11.1%)	1 (7.1%)
France	11	5	0	2	5	6	6	5	13	15	3	15	3	12	6	6	12
National	4 (36.4%)	0	0	0	2 (40.0%)	1 (16.7%)	0	0	4 (30.8%)	2 (13.3%)	2 (66.7%)	3 (20.0%)	1 (33.3%)	3 (25.0%)	1 (16.7%)	3 (50.0%)	1 (8.3%)
MR	3 (27.3%)	0	0	0	2 (40.0%)	1 (16.7%)	0	1 (20.0%)	2 (15.4%)	2 (13.3%)	1 (33.3%)	2 (13.3%)	1 (33.3%)	1 (8.3%)	2 (33.3%)	1 (16.7%)	2 (16.7%)
Centralized	4 (36.4%)	5 (100.0%)	0	2 (100.0%)	1 (20.0%)	4 (66.7%)	6 (100.0%)	4 (80.0%)	7 (53.8%)	11 (73.3%)	0	10 (66.7%)	1 (33.3%)	8 (66.7%)	3 (50.0%)	2 (33.3%)	9 (75.0%)
United Kingdom	11	5	0	2	1	10	5	5	13	16	2	15	3	11	7	6	12
National	6 (54.6%)	0	0	0	0	4 (40.0%)	0	1 (20.0%)	5 (38.5%)	4 (25.0%)	2 (100.0%)	4 (26.7%)	2 (66.7%)	3 (27.3%)	3 (42.9%)	4 (66.7%)	2 (16.7%)
MR	0	0	0	0	0	0	0	0	0	0	0	0	0	0	0	0	0
Centralized	5 (45.5%)	5 (100.0%)	0	2 (100.0%)	1 (100.0%)	6 (60.0%)	5 (100.0%)	4 (80.0%)	8 (61.5%)	12 (75.0%)	0	11 (73.3%)	1 (33.3%)	8 (72.7%)	4 (57.1%)	2 (33.3%)	10 (83.3%)
Germany	9	5	0	2	2	8	5	5	11	15	1	14	2	11	5	4	12
National	4 (44.4%)	0	0	0	1 (50.0%)	2 (25.0%)	0	1 (20.0%)	3 (27.3%)	3 (20.0%)	1 (100.0%)	3 (21.4%)	1 (50.0%)	3 (27.3%)	1 (20.0%)	2 (50.0%)	2 (16.7%)
MR	0	0	0	0	0	0	0	0	0	0	0	0	0	0	0	0	0
Centralized	5 (55.6%)	5 (100.0%)	0	2 (100.0%)	1 (50.0%)	6 (75.0%)	5 (100.0%)	4 (80.0%)	8 (72.7%)	12 (80.0%)	0	11 (78.6%)	1 (50.0%)	8 (72.7%)	4 (80.0%)	2 (50.0%)	10 (83.3%)

MA, market authorization; PE: patent expiry; MR, Mutual Recognition.

#### Designation change

A change in designation was observed in a minority of repositioning cases, with a higher ratio in Europe than in the United States (40.4% and 13.0% of the repositioning cases, respectively). In the United States, a designation change from non-orphan to orphan was mostly submitted as a new molecular entity (NDA type 1), whereas an sNDA (new indication) was the most frequent pathway if both the source and target product had the orphan designation. In Europe, the most frequent designation change was from non-orphan to orphan.

#### Approval time

Most of the repositioning cases in the United States were approved up to 2008 (82.6%). There was no clear trend on regulatory path used and year of approval, with a wide distribution of regulatory pathways per each year group. In Europe, most of the cases were approved after 1999, and most of the cases were filed as a centralized procedure.

#### Approval time after marketing authorization

Except for NDAs type 1, all repositioning cases were approved after marketing authorization of the source product in the United States. In Europe also, most cases were approved after marketing authorization of the source product, and in that situation the centralized procedure was the most frequently used pathway in all countries.

#### Patent expiry

In the United States, most repositioning cases (69.6%) were approved before the patent expiry of the original drug. Most cases approved before patent expiry were submitted as new molecular entities, new indications, or new dosage forms. In Europe, a similar pattern is seen regarding patent expiry and the regulatory pathway: the majority of repositioning cases were approved before patent expiry of the original product (83.3% in France, 88.9% in Germany, and 93.8% in the United Kingdom), and the most used regulatory pathway was through centralized procedure. All cases approved after patent expiry were approved by national procedure in all of the countries, with the exception of one case in France that was approved by the mutual recognition procedure.

#### Company

Most of the repositioned products were filed by the same company in the United States and in Europe (60.9% and 65.8% of cases in the United States and in Europe, respectively). In the United States, there was a heterogeneous distribution of the filing process used by the same company, with NDA type 1 section 505(b) and sNDA new indications accounting for most of the cases (64.7%). In Europe, the vast majority of cases filed by the same company were approved via centralized procedure (66.7%, 73.3%, and 78.6% in France, the United Kingdom, and Germany, respectively).

#### Brand name

56.0% of repositioning cases in the United States and 65.4% of the cases in Europe were filed with the same brand name. In the United States, most cases with the same brand name were filed as an NDA type 1 or as an sNDA (new indication), with most of the cases filed under a different brand name being filed under NDA type 3 (new dosage form). In Europe, most of the cases were filed under centralized procedure, with national procedures corresponding to the remaining processes (except in France, where mutual recognition was used in both situations).

#### Repositioning approach

In both the United States and Europe, a hypothesis-driven approach was followed for most of the cases (56.0% and 69.2%, respectively). In Europe, hypothesis-driven cases were more frequently filed under centralized procedures than serendipity approach cases, having these last shown a higher frequency of use of national procedure.

### Association between regulatory paths and attributes in reformulation cases

Table VII presents the distribution of the different regulatory application types and pathways according to their reformulation-related attributes, for each country.

**Table VII:** Distribution of regulatory application type by classification types within reformulation cases

	Designation change				Approval time			Marketing Authorization		Patent expiry		Company		Brand name		Reformulation group			
							
Application type	No change	Orphan drugs	Non orphan	Orphan to orphan	Before 1999	1999–2008	After 2008	Before MA	After MA	Before PE	After PE	Same company	Different company	Same brand	Different brand	Group 0	Group 1	Group 2	Group 3
United States	24	2	0	0	7	15	4	0	26	17	9	18	8	8	18	7	6	7	6
NDA type 1 section 505(b)	0	0	0	0	0	0	0	0	0	0	0	0	0	0	0	0	0	0	0
NDA type 2 section 505(b)	2 (8.3%)	0	0	0	0	2 (13.3%)	0	0	2 (7.7%)	1 (5.9%)	1 (11.1%)	1 (5.6%)	1 (12.5%)	0	2 (11.1%)	2 (28.6%)	0	0	0
NDA type 3 section 505 (b)	12 (50.0%)	2 (100.0%)	0	0	6 (85.7%)	7 (46.7%)	1 (25.0%)	0	14 (53.8%)	10 (58.8%)	4 (44.4%)	11 (61.1%)	3 (37.5%)	5 (62.5%)	9 (50.0%)	1 (14.3%)	4 (66.7%)	4 (57.1%)	5 (83.3%)
NDA type 3 section 505 (b)(2)	3 (12.5%)	0	0	0	1 (14.3%)	2 (13.3%)	0	0	3 (11.5%)	2 (11.8%)	1 (11.1%)	1 (5.6%)	2 (25.0%)	1 (12.5%)	2 (11.1%)	1 (14.3%)	1 (16.7%)	1 (14.3%)	0
NDA type 4 section 505 (b)	1 (4.2%)	0	0	0	0	1 (6.7%)	0	0	1 (3.8%)	0	1 (11.1%)	0	1 (12.5%)	0	1 (5.6%)	1 (14.3%)	0	0	0
NDA type 5 section 505(b)	1 (4.2%)	0	0	0	0	1 (6.7%)	0	0	1 (3.8%)	1 (5.9%)	0	1 (5.6%)	0	0	1 (5.6%)	1 (14.3%)	0	0	0
NDA type 5 section 505(b)(2)	2 (8.3%)	0	0	0	0	2 (13.3%)	0	0	2 (7.7%)	1 (5.9%)	1 (11.1%)	1 (5.6%)	1 (12.5%)	0	2 (11.1%)	1 (14.3%)	1 (16.7%)	0	0
NDA type 6 section 505(b)	0	0	0	0	0	0	0	0	0	0	0	0	0	0	0	0	0	0	0
sNDA (new indication)	0	0	0	0	0	0	0	0	0	0	0	0	0	0	0	0	0	0	0
sNDA (new formulation)	1 (4.2%)	0	0	0	0	0	1 (25.0%)	0	1 (3.8%)	0	1 (11.1%)	1 (5.6%)	0	0	1 (5.6%)	0	0	1 (14.3%)	0
sBLA	2 (8.3%)	0	0	0	0	0	2 (50.0%)	0	2 (7.7%)	2 (11.8%)	0	2 (11.1%)	0	2 (25.0%)	0	0	0	1 (14.3%)	1 (16.7%)
France	15	0	0	2	8	7	2	0	17	13	4	13	4	8	9	5	3	3	6
National	9 (60.0%)	0	0	0	7 (87.5%)	2 (28.6%)	0	0	9 (52.9%)	8 (61.5%)	1 (25.0%)	8 (61.5%)	1 (25.0%)	4 (50.0%)	5 (55.6%)	3 (60.0%)	3 (100.0%)	1 (33.3%)	2 (33.3%)
MR	3 (20.0%)	0	0	0	1 (12.5%)	2 (28.6%)	0	0	3 (17.6%)	1 (7.7%)	2 (50.0%)	1 (7.7%)	2 (50.0%)	0	3 (33.3%)	1 (20.0%)	0	0	2 (33.3%)
Centralized	3 (20.0%)	0	0	2 (100.0%)	0	3 (42.9%)	2 (100.0%)	0	5 (29.4%)	4 (30.8%)	1 (25.0%)	4 (30.8%)	1 (25.0%)	4 (50.0%)	1 (11.1%)	1 (20.0%)	0	2 (66.7%)	2 (33.3%)
United Kingdom	17	0	0	2	6	9	4	0	19	13	6	14	5	7	12	5	3	5	6
National	14 (82.4%)	0	0	0	6 (100.0%)	6 (66.67%)	2 (50.0%)	0	14 (73.7%)	9 (69.2%)	5 (83.3%)	10 (71.4%)	4 (80.0%)	3 (42.9%)	11 (91.7%)	4 (80.0%)	3 (100.0%)	3 (60.0%)	4 (66.7%)
MR	0	0	0	0	0	0	0	0	0	0	0	0	0	0	0	0	0	0	0
Centralized	3 (17.6%)	0	0	2 (100.0%)	0	3 (33.3%)	2 (50.0%)	0	5 (26.2%)	4 (30.8%)	1 (16.7%)	4 (28.6%)	1 (20.0%)	4 (57.1%)	1 (8.3%)	1 (20.0%)	0	2 (40.0%)	2 (33.3%)
Germany	10	0	0	2	3	7	2	0	12	11	1	10	2	6	6	4	1	3	4
National	7 (70.0%)	0	0	0	3 (100.0%)	4 (57.1%)	0	0	7 (58.3%)	7 (63.6%)	0	6 (60.0%)	1 (50.0%)	2 (33.3%)	5 (83.3%)	3 (75.0%)	1 (100.0%)	1 (33.3%)	2 (50.0%)
MR	0	0	0	0	0	0	0	0	0	0	0	0	0	0	0	0	0	0	0
Centralized	3 (30.0%)	0	0	2 (100.0%)	0	3 (42.9%)	2 (100.0%)	0	5 (41.7%)	4 (36.4%)	1 (100.0%)	4 (40.0%)	1 (50.0%)	4 (66.7%)	1 (16.7%)	1 (25.0%)	0	2 (66.7%)	2 (50.0%)

MA, market authorization; PE, patent expiry; MR, Mutual Recognition.

#### Designation change

Only a very small number of reformulation target cases have received an orphan designation (7.69% cases in the United States and none in Europe, excluding the 14.1% of cases that kept the orphan designation already given to the source product). Cases classified as orphans followed an NDA type 3 (B) application in the United States and the centralized procedure in Europe.

#### Approval time

In the United States, most of reformulation cases were approved in the period from 1999 to 2008. Before 1999, the cases were filed exclusively as NDA type 3 applications, and in the period after 2008, they were filed as NDA type 1, sNDA (new formulation), and SBLA. In Europe, most of the reformulation cases approved before 1999 were approved under the centralized procedure.

#### Approval time after Market Authorization

All reformulation target cases were approved after marketing authorization of the source product in the United States and in Europe.

#### Patent expiry

65.3% of reformulation cases in the United States were approved before patent expiry, most of these being filed as an NDA type 3 section 505(b). Also in Europe, most of the reformulation cases were approved before patent expiry (77.1%), with a higher use of national procedure before patent expiry in all European countries.

#### Company

Most of the reformulated cases in the United States were submitted by the same company (69.2%), and the majority followed an NDA type 3 section 505(b) pathway. In Europe, there is also a clear trend for reformulation cases being developed by the same company (77.1% of cases), with most of those cases being submitted through national procedure in all of the countries.

#### Brand name

In the United States, most of the reformulation cases have a different brand name from the original product (69.2%), with most of these cases following an NDA type 3 section 505(b) pathway. Reformulations approved with a different brand name in Europe were mostly approved through national procedure.

#### Reformulation group

Most of the reformulation cases corresponding to Group 1, Group 2, and Group 3 in the United States have been approved as an NDA type 3 section 505(b). For Group 0 reformulations in the United States, the cases were distributed between type 2 section 505(b) and section 505(b)(2), type 3 section 505(b), type 4 section 505(b), and type 5 section 505(b). In Europe, Group 0 reformulations were most frequently approved through national procedure, and all Group 1 reformulations were approved through national procedure. As for Group 2 and Group 3 reformulations, there is not a clear trend regarding the regulatory process used.

## Discussion

The evaluation of the distribution of regulatory paths for the repurposing case studies confirms the importance of the regulatory framework in shaping the development of drug repurposing, particularly for drug reformulations and repositioning.

Regulatory framework changes, such as the Hatch–Waxman Act in the United States, had a direct, albeit slow, impact on repurposing development strategies. In fact, with the section 505(b)(2) pathway, regulators in the United States encouraged pharmaceutical companies to find innovative purposes for their developed drugs by allowing developers to reference known information on approved drugs to create differentiated products. It is reported that from the passage of the act in 1984 to 1998, only 10 drug approvals were based on the 505(b)(2) pathway (15). It was only after 2002 that developers increased their interest in this regulatory path, so much so that half of the approved drugs in the United States in 2008 were approved via this regulatory procedure.

From our analysis of the different repurposing cases, it appears that developers have multiple options regarding regulatory application types either in the United States or in Europe. For the United States, in addition to the different pathways of section 505 that a developer can follow, several NDA chemical types can apply for the same repurposing type. Chiral switch (classified under reformulation Group 0 according to our taxonomy) can be an illustrative example; in fact, in the United States, for chiral switches the FDA assigns chemical types on a case-by-case basis, which explains why for some cases such as levalbuterol and levofloxacin, the new target products were classified as new formulations (NDA type 3), and for others such as esomeprazole and levobupivacaine the new target products were considered as new derivatives of an already marketed active ingredient (NDA type 2).

The results confirmed the appropriateness of our definitions of repositioning and reformulations: this is illustrated by the use of NDA chemical types 1 and 6 and sNDA (new indication), which are solely for drug-repositioning applications, and type 5(2) and sNDA (new formulation), which are only for reformulations. Also, most of the reformulation cases being approved as NDA chemical types 3, and 5 (i.e. new dosage form or new formulation, respectively) corroborate the previous findings.

For Europe, it was not possible to retrieve the information on which regulatory basis applications for repurposed products were submitted. However, it is known that articles 10(3), 10a, 8(3), and 6 of Directive 2001/83/EC partially cover different repurposing scenarios (13, 15). Those regulations are not equally used by developers; an analysis showed that repositioning developers often submitted their drug registrations for repurposing through the use of article 8(3) and submitted full dossiers rather than submitted hybrid applications according to article 10(3), which provides a regulatory framework for new indications for existing approved drugs (16).

It was not possible to establish a correlation between the regulatory application types used in the United States and in Europe per type of repurposing strategy. In fact, the regulatory application types in the United States and in Europe follow different criteria for selection of the cases (in the United States it is mostly a function of the product's individual characteristics, whereas in Europe there is an articulation between the therapeutic indication and the type of application). The regulatory framework for drug applications in Europe had two major changes in the 1990s and 2000s: it changed in 1993 with the introduction of the centralized and mutual recognition procedure, and it changed later on in 2004 with the introduction of the decentralized procedure and new criteria for the centralized procedure. This fact also may contribute to the diversity of application types in Europe corresponding to the same application type in the United States.

Within the studied European countries, most of the applications for reformulated products were filed on a national level. In contrast, for repositioned products, the centralized procedure was more frequently used as the regulatory pathway. Interestingly within the cases studied, the mutual recognition procedure was only used in France and was evenly used for reformulation and repositioning cases. Indeed we verified that in Europe, reformulations and repositioning follow distinct and almost opposite regulatory trends: most cases of repositioning were approved through centralized procedure, with only a minority being approved through national procedure, and the inverse was observed for reformulations. It seems that repositionings are developed and processed in Europe as entirely new drug applications, whereas reformulations are managed mostly as abridged applications, corroborating the concept of ‘line extension’.

Our results also showed that in the United States, certain application pathways are exclusively related with drug repositioning [NDA chemical type 1 (new molecular entity), type 6 (new indication), and sNDA (new indication)] or with drug reformulations [NDA chemical type 2 (change in active ingredient), type 5(2) (new formulation or new manufacturer), and sNDA (new formulation)].

As for designation change (or maintenance of orphan status), the results are in line with the regulatory requirements of mandatory filling of these products under centralized procedure in Europe and under O classification in the United Status.

The totality of reformulations being approved after the market authorization of the source product confirms the accuracy of the proposed taxonomy. As for US repositioning cases, the appropriateness of this taxonomy was also confirmed, with all the cases approved after the market authorization of the source drug being filed as NDA type 1 (new chemical entities).

Our results support that most of the repurposing cases are approved before patent expiry of the source product. The repositioning cases approved before patent expiry have followed more complex regulatory pathways in the United States and in Europe. This can be related to the pursue of longer exclusivity periods by drug developers. As for reformulations, although most cases were also approved after patent expiry in all countries, there was not a trend for use of a complex regulatory path. These results demonstrate a clear distinction in the regulatory pathway between repositioning and reformulations and approval before patent expiry.

Most of the repurposed cases in the United States were submitted by the same company that submitted the original product, with a similar trend observed in Europe. It seems, however, that in the United States this is not a parameter that has an impact on predetermining the type of regulatory pathway used, as no clear trend was observed. As for Europe, most companies follow a centralized procedure for repositioning irrespective of which company filed the original molecule, which again can indicate that ‘company filling’ is not a parameter that predetermines the regulatory path.

When using the same brand name, the repositioned product was mainly filed as a new molecular entity or a new indication in the United States, whereas most cases filed under a different brand name followed an NDA type 3 section application, which means a change in the dosage form. Hence, the use of a different brand name in these situations may be due to a need to provide a differentiation for the product. In Europe, repositioning cases were also more frequently approved under the same brand name, but there is no clear trend for reformulations. This can be explained in Europe by the mixed use of regulatory pathways for reformulations which determine the possibility of keeping the same brand name.

Our results show that for new molecular entities filed in the United States, a similar number of cases were developed by serendipity and by a hypothesis-driven approach. However, for the regulatory pathway of new indications, most of the cases are developed through a hypothesis-driven approach. This can be explained by the recent and systematic efforts of developers to find new pharmacological targets for existing drugs in their portfolio. As for Europe, there is a clear trend for centralized approval of repositioned cases developed by a hypothesis-driven approach, which can again be explained by the use of systematic and carefully planned efforts when using that approach, which is necessary for a complex pathway such as the centralized procedure.

Lastly, results confirmed the robustness of our nomenclature and taxonomy for drug reformulations: all cases of reformulation Group 1, Group 2, and Group 3 in the United States have been approved as new dosage forms, reformulations or as minor changes (new dosage and strength) to the BLA. This was also the case for cases classified as Group 0 i.e. chiral switches and other switches, except that one-third of those were classified as ‘chemical derived from an active ingredient already marketed’, which is completely aligned with our definition of Group 0 (1).

Drug repurposing is closely associated with intellectual property (IP) matters, and developers have to be well prepared to face IP-related challenges (17). In the United States, obtaining composition-of-matter (COM) and use patents is possible with drug repurposing (17). The strongest patent protection can be offered by COM patents typically covering the API. This was the case for sildenafil citrate when it was repositioned from angina (its initially intended indication) to erectile dysfunction, or for crizotinib, which was repositioned from an initially intended indication in anaplastic large-cell lymphoma to the actual indication approved in non-small cell lung cancer (NSCLC) (18, 19). Patents covering formulations and delivery mechanisms come in second place in terms of strength of patent protection. For example, the transdermal patch of rivastigmine has been covered by a delivery mechanism patent (20). One of the best scenarios for use patents is when the API for a drug-repositioning case is not already on the market and the new product is only approved for the new use (14).

Compared to the United States, the ‘method-of-use’ patents are weaker in Europe, where patent term extensions for already approved drugs are less likely to be issued (16). In contrast, as illustrated in Table II, developers can receive higher incentives for orphan drugs and pediatric populations in Europe than in the United States. In a similar analysis of the regulatory framework for drug repositioning, European authorities seem to be less supportive and more restrictive toward drug repositioning than the American authorities (16).

In order to enable geographical comparisons between cases and eliminate bias of the multiplicity of strategies used for some cases, we have selected those cases that were approved in all countries and that have incurred only one type of repurposing strategy. Although this selection process provides a more clear interpretation, it eliminated more complex cases, and as such we cannot fully extrapolate our results to those situations. Also, due to our selection criteria, not all classification types were covered by our case studies, such as repositioning cases via a non-hypothesis-driven approach (which are still in clinical trials), off-target repositioning of cases in the same therapeutic area as the source product (rare cases), among others.

Another limitation is the wide difference in data availability between the studied countries. In the United States, we could access approval letters and review folders that are available in the FDA database under the provisions of the Freedom of Information (FOI) Act. It has to be noted that such data are less likely to be available for products approved prior to 2000. In Europe, despite data searches on regional and national levels, detailed information on application dossiers for all of the studied repurposing cases could not be retrieved. Thus, our regulatory analysis for European countries was limited to approval dates and the application process used (e.g., centralized, national or mutual recognition/decentralized).

Lastly, detailed information on exclusivity and IP protection, which could not be retrieved for the studied cases, could have been an interesting angle from which to evaluate the benefits in terms of protection period that repurposing strategies can potentially offer in Europe and the United States.

There have been recent initiatives to encourage information sharing and partnering for repurposing among academic researchers (from public centers and universities) and pharmaceutical companies (17, 21, 22). The most recent of these initiatives have been launched by the National Institutes of Health (NIH) National Centre for Advancing Translational Sciences (NCTAS) for drug repurposing in the United States and by the United Kingdom's Medical Research Council Initiative (MRC) (23). These programs provide legal and administrative processes for partnering across multiple organizations, including the IP responsibility split. It can be expected that through these open access initiatives, the number of molecules that are screened and eventually repurposed will dramatically increase in the future.

## Conclusion

Over time, regulatory changes in the United States and Europe have led to a trend to encourage drug developers to strive for innovation, and also more or less explicitly to strive for repurposing existing or known drugs. This applies to all repurposing strategies, which have certain differences in terms of regulatory paths, application types, and required evidences, which are relatively specific and established on a case-by-case basis. It is also seen that within drug repurposing, drug reformulation has been increasingly regulated in Europe.

Specific regulations and detailed guidelines exist for some drug-repurposing situations. Additionally, the incentives that a repurposed product can receive in terms of patent extension and exclusivity period protection make such strategies particularly attractive. Repositioning a non-orphan drug for an orphan disease is an excellent example of an appealing development, with relatively easier regulatory requirements and some interesting economic and exclusivity incentives.

The regulations in the United States and in Europe for drug repositionings and drug reformulations allowed confirming that repositioning strategies are usually filed under a more complex regulatory process than reformulations. There are regulatory pathways in the United States and in Europe that are used exclusively for drug repositionings or for drug reformulations. Also, we have not found a correlation between individual regulatory pathways in the United States and in Europe.

Certain parameters, such as patent expiry and type of repositioning approach or reformulation type, are also conditioners of the regulatory pathways chosen for each case.

Given all the regulatory encouragements and restrictions by regulators and health authorities and the growing pressure of increasing healthcare expenditures, bringing a real added value to society is a must to convince key stakeholders, obtain market access, and successfully reach the market.

In the last study of this series of three publications, we will evaluate which parameters are considered to be linked to an added-value perception and identify the determinants of successful market access outcome for repurposed drugs.
